# Health Care Consumption during Pregnancy in relation to Maternal Body Mass Index: A Swedish Population Based Observational Study

**DOI:** 10.1155/2015/215683

**Published:** 2015-05-26

**Authors:** Elisabeth S. Lindholm, Daniel Altman, Margareta Norman, Marie Blomberg

**Affiliations:** ^1^Division of Obstetrics and Gynecology, Department of Clinical Science, Karolinska Institutet, 171 77 Stockholm, Sweden; ^2^Department of Obstetrics and Gynecology and Department of Clinical and Experimental Medicine, Linköping University, 581 83 Linköping, Sweden

## Abstract

*Objective*. To assess whether antenatal health care consumption is associated with maternal body mass index (BMI). *Design*. A register based observational study. *Methods*. The Swedish Medical Birth Register, the Maternal Health Care Register, and the Inpatient Register were used to determine antenatal health care consumption according to BMI categories for primiparous women with singleton pregnancies, from 2006 to 2008, *n* = 71,638. Pairwise comparisons among BMI groups are obtained post hoc by Tukey HSD test. *Result*. Obese women were more often admitted for in-patient care (*p* < 0.001), had longer antenatal hospital stays (*p* < 0.001), and were more often sick-listed by an obstetrician (*p* < 0.001) during their pregnancy, compared to women with normal weight women. Preeclampsia was more than four times as common, hypertension five times as common, and gestational diabetes 11 times as common when comparing in-patient care, obese to normal weight women (*p* < 0.001 for all comparisons). Underweight mothers had longer stay in hospitals (*p* < 0.05) and hydronephrosis and hyperemesis gravidarum were more than twice as common (both *p* < 0.001). *Conclusion*. Obese and underweight mothers consumed significantly more health care resources and obese women were significantly more often sick-listed during their pregnancy when compared to pregnant women of normal weight.

## 1. Introduction

The incidence of obesity is increasing worldwide and at present about 12.6% of Swedish pregnant women are obese, as compared to 20.2% of women in childbearing age in the UK [1] and 26.5% in US women, ages: 20 to 39 [2]. Excess bodyweight is the sixth most important risk factor contributing to the overall burden of disease worldwide [3] and has been shown to decrease life expectancy in women by 7 years [4]. It is already known that maternal overweight and obesity, as measured by body mass index (BMI), are associated with adverse obstetric and neonatal outcomes [5, 6]. During pregnancy obese women have an increased risk for preeclampsia [7], hypertension [7], gestational diabetes [8], preterm birth [9], and cesarean section [10]. A previous study from the United States showed that obesity during pregnancy was associated with increased use of health care services especially related to the rate of cesarean section and obesity-related high-risk conditions [11]. A study from Wales showed a strong association between healthcare usage cost and BMI [12].

The objective of the present study was to determine to which extent pregnant women in different BMI classes in Sweden consume in- and outpatient healthcare resources during pregnancy compared to women with normal weight using nationwide data from three medical health care registers.

## 2. Materials and Methods

This is a population-based cohort study including 88,120 primiparous women with singleton births recorded in the Swedish Medical Birth Register (MBR) from January 1, 2006, through December 31, 2008. Using the unique national registration number assigned to all Swedish residents, the MBR was linked with two other national registers: the Maternal Health Care Register and the Inpatient Register.

Swedish prenatal care takes place within the primary health care system, mainly in outpatient clinics, with a midwife as the primary caregiver, and is free of charge for all Swedish residents. At the first prenatal visit, normally at 8–12 weeks of gestation, information on maternal demographic data, health care history and reproductive history, and self-reported maternal height and weight measured (from which BMI (kg/m^2^) is calculated) is collected by a midwife. The standardized records are identical throughout the country and are forwarded to the MBR where the information is computerized. The MBR has recorded data about more than 98% of all deliveries in Sweden since 1973 and has previously been validated and described in detail [13].

The Maternal Health Care Register (MHCR) was created in 1999 as a complementary data base to the MBR in order to measure antenatal health care quality. A number of variables not available in the MBR were thus selected and included in the MHCR. Midwives report data twice to the Maternal Health Care Register, at the beginning of the pregnancy and 16 weeks after delivery. Most of the registered items entered in the MHCR are data obtained from medical records manually registered by a midwife. The study time 2006–2008 was chosen because the question about maternal sick-leave during pregnancy was reported exclusively during these years. The MHCR has recently been validated [14]. There was a national coverage of 75–77% of all pregnancies in Sweden between 2006 and 2008.

Out of the 88,120 primiparous women with singleton births recorded in the MBR a total number of 78,263 could be linked to the MHCR (88.8%).

Exposure in this study was maternal body mass index (BMI). Women were grouped into five BMI categories: underweight <20; normal weight (20–<25); overweight (25–<30); obese (30–<35); and morbidly obese (≥35). In the study population of 78,263 primiparous women with singleton births and available data sets in both the MBR and the MHCR, there were 6,625 (8.5%) women where maternal BMI could not be calculated due to missing values which enables a final study population of 71,638.

Outcome evaluated was maternal health care consumption in the antenatal period up till the day before the delivery and it included the following variables and parameters: the number of visits to a physician at the maternal health care centre; the number of visits to a midwife at the maternal health care centre; the number of visits to cross-disciplinary clinics for treatment of mothers having moderate to severe fear of childbirth (referred to as Aurora clinics in Sweden); and sick-listing during pregnancy.

Another way of measuring maternal health care consumption in the antenatal period was to estimate the number of hospital admissions and the number of days admitted to hospital and to evaluate the causes of the inpatient care. For this purpose the Swedish Inpatient Register (IPR) was used. The IPR has complete national coverage from 1987 and more than 99% of all somatic and psychiatric hospital discharges are registered. A previous validation of the IPR by the National Board of Health and Welfare showed that 85–95% of all diagnoses in the IPR were valid [15]. The diagnoses are coded according to the International Classification of Diseases (ICD-10 since 1997).

The study was approved by the Stockholm Regional Board of Ethics at Karolinska Institute, Stockholm, Sweden, dnr: 2008/1427-31/3.

## 3. Statistics

All statistics were performed in *R*, version 2.14.1. For numerical variables means and standard deviations are presented, whereas one-way ANOVA was used for testing the hypothesis of equal means. Pairwise comparisons among groups are obtained post hoc by the Tukey HSD test. For categorical variables frequencies and percentages are presented. Where frequencies are presented, the *p* overall in the following tables is from the *R* function probability test, a test of equal proportions. Also presented are *p* values for pairwise comparisons among all BMI groups: these are Holm adjusted post hoc tests by *R* function pairwise probability test. The results were corrected for smoking.

## 4. Results

The final study population consisted of 71,638 primiparous women with singleton births and data on maternal BMI in early pregnancy and available data sets in both the MBR and the MHCR.

The prevalence of underweight (BMI < 20) was 11.7%, overweight (BMI 25–29.9) 22.9%, obesity (BMI 30–34.9) 6.8%, and morbid obesity (BMI ≥ 35) 2.9%. Maternal characteristics and maternal morbidities according to BMI class are presented in Table 1. Obese women were older, smoked to a larger extent, and had more often asthma, pregestational hypertension, and diabetes mellitus compared to normal weight women.

Table 2 shows the health care consumption during pregnancy in each maternal BMI class, both the outpatient care at the prenatal clinic and the inpatient care at the hospital. There was a small increase in the number of visits to both obstetrician and midwife with increasing BMI category. Obese and morbidly obese women had statistically significantly more visits to both obstetrician and midwife compared to normal weight mothers. Morbidly obese women had on average 0.9 more visits to the midwife and 0.5 more visits to the physician compared to normal weight women. Women underweight had more visits to doctors but less visits to their midwives during pregnancy compared to normal weight women. The number of women referred to an Aurora clinic increased with increasing BMI and was significantly more common among obese and morbidly obese women compared to normal weight and overweight mothers (*p* < 0.001 for all comparisons). The number of sick-listed women during pregnancy increased significantly with increasing BMI.

Obese women and morbidly obese women were significantly more often admitted to hospital during pregnancy and had a longer average in-hospital stay compared to normal weight and overweight mothers. Stratifying for maternal smoking during pregnancy did not change any results in Table 2 (data not shown).

In-patient care during pregnancy and related diagnoses in each maternal BMI category are shown in Table 3. The five most common diagnoses for in-patient care during pregnancy were pregnancy induced abdominal pain (0.73%); premature contractions without cervical ripening (0.69%); imminent premature labor (0.68%); severe hyperemesis gravidarum (0.66%); and other bleeding before pregnancy (0.64%). However, none of these diagnoses showed a positive association with neither overweight nor obesity. On the contrary, being overweight and obese significantly reduced the in-patient care because of imminent premature labor (*p* < 0.001 for both BMI categories) in comparison with normal weight women. The prevalence of in-patient care of pregnancy induced hypertension, preeclampsia, and gestational diabetes was, however, increased among obese and morbidly obese women as compared to normal weight women (*p* < 0.001 for all comparisons). In-patient care for preeclampsia was more than four times as common, in-patient care for hypertension five times as common, and in-patient care gestational diabetes 11 times as common when comparing obese to normal weight women (*p* < 0.001 for all comparisons). In-patient care for hydronephrosis was twice as common and in-patient care for severe hyperemesis gravidarum was nearly three times as common in underweight mothers compared to normal weight women (*p* < 0.001 for both comparisons).

Because tobacco smoking was nearly twice as common before and during pregnancy among obese women, compared to normal weight women (Table 1), we performed a complementary analysis of the causes of in-hospital care, from which smokers before and during pregnancy were excluded. This analysis yielded essentially unaltered patterns in pregnancy-related morbidity over the BMI strata (data not shown).

## 5. Discussion

This large population-based cohort study based on three Swedish medical health registers showed that obese women generally consumed more health care resources during pregnancy compared to normal weight women. Obese women were more often admitted for in-patient care, had longer antenatal hospital stays, and were more often sick-listed during their pregnancy, compared to normal weight women. The differences were all statistically significant but in absolute numbers not so impressive.

Another finding was that obese women were less likely to be hospitalized for imminent premature labor, that is, childbirth before gestational week 37. There may be several reasons for this, including misdiagnosis of premature contractions which might be more difficult to diagnose correctly in obese women. Possibly obese women are less inclined to seek care, or they may perceive contractions differently. Whether maternal obesity is a risk factor for spontaneous preterm birth is conflicting. Cnattingius et al. [9] showed in a large population-based study higher risk for spontaneous extremely preterm delivery for obese women, but in the study by Hendler et al. [16] they found lower incidence of spontaneous preterm birth in obese mothers.

Part of the increased number of visits to health care providers was likely related to somatic pregnancy complications and obese women had higher rates of hypertension, preeclampsia, and diabetes. Whether the greater number of visits to Maternal Health Care Centers was related to worry and anxiety among obese pregnant women is a matter of speculation. This notion is indirectly supported by the significantly higher occurrence of referrals to Aurora units for prenatal counseling of fear of childbirth among obese. Obese women are known to experience feelings of anxiety and worry related to the upcoming birth to a higher degree than normal weight women [17]. Underweight mothers also consume more healthcare resources mainly because of longer stay in hospitals and more visits to doctors. It seemed that the main reason for that was due to hyperemesis gravidarum which is in accordance with earlier studies [18].

The strengths of this study are the size of the study population, including over 2000 morbidly obese women, and further the use of nationally uniform outcome measures and the availability of validated high quality health care registers. Although much attention has been devoted to morbidity and complications surrounding delivery, this is one of the few studies focusing on antenatal health care consumption among obese women and the first one in the Swedish context. The unique national registration number assigned to all Swedish residents, at birth or immigration, allowed for unambiguous record linkage across the registers, as well as minimizing selection and ascertainment bias. Among the limitations of our study it should be noted that the MHCR did not have nationwide coverage at the time of the study and that regional variations in demographics may have influenced the available data and thereby results. Nonetheless, the register includes both urban and rural populations and has a geographical spread throughout the country's population, which is relatively homogeneous.

The incidence of smoking before pregnancy, at the first antenatal visit, and three months into pregnancy increased significantly with increasing BMI. Among the morbidly obese 10% admitted that they were still smoking in gestational weeks 30–32 despite mandatory counseling and information by midwives at the Maternal Health Care Centers. As such, smoking does not appear to confound the association between the increased prevalence of hypertension, preeclampsia, and gestational diabetes with increasing BMI. Many studies have showed that smoking during pregnancy reduces the risk of preeclampsia and gestational hypertension by up to 50% [19]. However, the prevalence estimates of these cardiovascular diseases were practically unaffected by the exclusion of smoking women in a separate analysis. There might have been several reasons for this. First, it is possible that the mechanisms underlying the protective effects of smoking on the risk of preeclampsia among obese women differ from the corresponding mechanisms among normal weight women. Second, the numbers of smokers with preeclampsia and hypertension were low, and exclusion of these women did not have a significant impact on the occurrence of disease. Third, an unknown confounder may have interfered with the analysis.

Few studies have reported on obesity-related consumption of health care services during pregnancy. Two studies from Montpellier, France, from 1980 to 1993 and 1993 to 1994 [20, 21] showed that the average cost for in-patient care was significantly higher for overweight and obese women than for normal weight women. Chu et al. [11] showed, using data from the Northwestern United States 2000–2004, that BMI was associated with significantly more prenatal tests and prenatal visits to physicians. In these studies, most of the increased hospital stay durations among obese women were related to increased rates of caesarean delivery and obesity-related high-risk conditions at delivery [11]. The study, however, shows that obesity increases the rate of hospital admissions and length of stay prior to delivery as well. Obesity is thus a source of economic burden for antenatal health care, as well as for hospital care. Moreover, Stafne et al. showed that the proportion of women sick-listed due to lumbar-pelvic pain was lower in a group of women undertaking aerobic and strengthening exercises during pregnancy, compared to women given standard antenatal care [22]. Back pain is more common in obese women than in normal weight women and increases with age [23], which may yield additional health care benefits from an interventional program for the former group.

In Sweden, the social welfare system allows for sick-listing by a physician in cases of transient or permanent disability caused by disease, in which case the cost and loss of income are partly reimbursed by the state. In our study, one in three obese women was reported to have been sick-listed during pregnancy increasing in a near-linear fashion with increasing BMI. Given that approximately one in ten pregnant women in Sweden are obese, it is safe to say that society's health care costs for sick-listing attributed to obesity are considerable. Since we had no access to detailed information on sick-listing diagnoses or duration, the extent of inability to work and the association with obesity requires further studies.

Obese women visited both midwives and doctors at Maternal Health Care Centers statistically significantly more often than normal weight women. Given that obesity during pregnancy is considered a risk for a number of complications, it is somewhat surprising that the difference was not even greater. While this finding is of importance for allocating health care resources, it also highlights the need for prevention. It has been shown that rates of preeclampsia, caesarean section, instrumental delivery, and babies large for gestational age can be reduced in obese women with low weight gain during pregnancy compared to women with high gestational weight gain [24, 25]. It has also been demonstrated that weight gain during pregnancy among obese women can safely be restricted through soft interventional programs including dietary advice, physical exercise, and supportive counseling and monitoring by dedicated staff [26, 27] but there is urgent need for more studies showing the best way to prevent heavy weight gain during pregnancy, especially for obese women. However, the complexity of intervention studies makes it necessary to evaluate the fidelity of the intervention itself in addition to its outcomes [28]. Given the major health economic and medical consequences of pregnancy in overweight and obese women, all attempts should be made to prevent obesity in women of childbearing age and to encourage weight loss before pregnancy.

## 6. Conclusion

Obese and underweight mothers consumed significantly more health care resources and obese women were significantly more often sick-listed during their pregnancy when compared to pregnant women of normal weight.

## Figures and Tables

**Table 1:** Maternal characteristics and morbidities.

		<20 (*N* = 8, 392)	20–<25 (*N* = 40, 220)	25–<30 (*N* = 16, 041)	30–<35 (*N* = 4, 902)	35+ (*N* = 2, 083)	Total (*N* = 71, 638)
Age	Mean (SD)	27.1 (4.9)^*∗∗*^	28.3 (4.9)	28.4 (5.2)	28.1 (5.2)^*∗∗*¶¶^	28.3 (5.2)	28.1 (5.0)

Smoking three months before pregnancy	*n* (%)	1676 (20.0%)^*∗∗*^	7290 (18.1%)	3625 (22.6%)^*∗∗*^	1319 (26.9%)^*∗∗*¶¶^	615 (29.5%)^*∗∗*¶¶#^	14525 (20.3%)
Smoking at admission to Maternal Health Care Center	*n* (%)	672 (8.0%)^*∗∗*^	2229 (5.5%)	1189 (7.4%)^*∗∗*^	496 (10.1%)^*∗∗*¶¶^	269 (12.9%)^*∗∗*¶¶#^	4855 (6.8%)
Smoking, during gestational weeks 30–32	*n* (%)	456 (5.4%)^*∗∗*^	1492 (3.7%)	857 (5.3%)^*∗∗*^	372 (7.6%)^*∗∗*¶¶^	215 (10.3%)^*∗∗*¶¶##^	3392 (4.7%)
Asthma and other lung diseases	%	7.24%	7.58%	9.43%^*∗∗*^	11.77%^*∗∗*¶¶^	13.78%^*∗∗*¶¶#^	8.42%
Ulcerative colitis/Crohn's disease	%	0.66%	0.70%	0.75%	0.67%	0.82%	0.70%
Epilepsy	%	0.54%	0.48%	0.62%^*∗*^	0.67%	0.62%	0.54%
Chronic kidney disease	%	0.48%	0.37%	0.46%	0.51%	0.38%	0.41%
Pregestational hypertension	%	0.21%	0.23%	0.51%^*∗∗*^	0.84%^*∗∗*¶^	0.96%^*∗∗*¶^	0.35%
Diabetes mellitus	%	0.08%^*∗*^	0.20%	0.52%^*∗∗*^	0.43%^*∗*^	0.96%^*∗∗*¶#^	0.30%
Systemic lupus erythematosus (SLE)	%	0.10%	0.09%	0.09%	0.12%	0.05%	0.09%

*∗* denotes significant difference between BMI between 20 and <25, ¶ between 25 and <30, and # between 30 and <35. Single legends denote significance at level <0.05 and double legends denote significance at the <0.001 level.

**Table 2:** Health care consumption during pregnancy.

BMI		<20	20–<25	25–<30	30–<35	35+	Total
*n*		8,392	40,220	16,041	4,902	2,083	71,638
Mean number of visits to physician at maternal health care centre (SD)	Mean (SD)	3.3 (1.7)^*∗*^	3.2 (1.6)	3.4 (1.8)^*∗∗*^	3.5 (1.9)^*∗∗*¶¶^	3.8 (2.1)^*∗∗*¶¶##^	3.3 (1.7)
Mean number of visits to midwife at maternal health care centre (SD)	Mean (SD)	10.8 (2.4)^*∗∗*^	10.9 (2.3)	11.0 (2.6)^*∗∗*^	11.4 (2.9)^*∗∗*¶¶^	11.8 (3.2)^*∗∗*¶¶##^	10.9 (2.4)
Number of women sick-listed during pregnancy (%)	*n* (%)	1925 (22.9%)	8977 (22.3%)	4250 (26.5%)^*∗∗*^	1470 (30.0%)^*∗∗*¶¶^	718 (34.5%)^*∗∗*¶¶##^	17340 (24.2%)
Number of women referred to Aurora clinic *n* (%)	*n* (%)	386 (4.6%)	1631 (4.1%)	706 (4.4%)^*∗∗*^	238 (4.9%)^*∗∗*¶¶^	122 (5.9%)^*∗∗*¶¶^	3083 (4.3%)
Mean number of hospital admission (SD)	Mean (SD)	0.3 (0.7)^*∗*^	0.3 (0.6)	0.4 (0.7)^*∗∗*^	0.4 (0.7)^*∗∗*¶¶^	0.5 (0.8)^*∗∗*¶¶##^	0.3 (0.6)
Mean number of days admitted to hospital (SD)	Mean (SD)	0.9 (4.0)^*∗*^	0.8 (2.8)	1.0 (2.9)^*∗*^	1.2 (3.1)^*∗∗*¶¶^	1.7 (3.8)^*∗∗*¶¶##^	0.9 (3.0)

*∗* denotes significant difference between BMI between 20 and <25, ¶ between 25 and <30, and # between 30 and <35. Single legends denote significance at level <0.05 and double legends denote significance at the <0.001 level.

An Aurora clinic is a cross-disciplinary unit for treatment of moderate to severe fear of childbirth.

**Table 3:** Most common diagnoses for inpatient care hospital admissions in the entire study population.

BMI	<20	20–<25	25–<30	30–<35	35+	Total
*n* =	8,392	40,220	16,041	4,902	2,083	71,638
	%	%	%	%	%	%
Abdominal pain	0.83%	0.68%	0.72%	1.04%^*∗*¶^	0.62%	0.73%
Contractions without cervical ripening before pregnancy week 37	1.22%	0.65%	0.55%	0.55%	0.58%	0.69%
Threatening premature labour (< pregnancy week 37)	1.10%^*∗∗*^	0.69%	0.50%^*∗*^	0.43%^*∗*^	0.58%	0.68%
Severe hyperemesis gravidarum	1.08%	0.62%	0.57%	0.49%	0.82%	0.66%
Other bleeding before delivery	0.66%	0.67%	0.60%	0.43%	0.62%	0.64%
Preeclampsia, mild to moderate	0.23%	0.39%	0.87%^*∗∗*^	1.29%^*∗∗*¶^	2.16%^*∗∗*¶¶#^	0.59%
Care due to breech position	0.37%	0.33%	0.39%	0.41%	0.29%	0.35%

Pregnancy induced hypertension	0.10%	0.19%	0.45%^*∗∗*^	0.71%^*∗∗*¶^	1.30%^*∗∗*¶¶#^	0.31%
Other specific pregnancy conditions	0.20%	0.24%	0.24%	0.37%	0.62%^*∗*¶^	0.25%
Pyelonephritis	0.27%	0.24%	0.21%	0.18%	0.19%	0.23%
Hydronephrosis	0.38%^*∗∗*^	0.17%	0.19%	0.10%	0.05%	0.19%
Cystitis during pregnancy	0.18%	0.15%	0.12%	0.12%	0.24%	0.15%
Hyperemesis gravidarum, unspecified	0.32%^*∗∗*^	0.11%	0.11%	0.06%	0.10%	0.13%
Mild hyperemesis gravidarum	0.30%^*∗∗*^	0.10%	0.13%	0.04%	0.19%	0.13%
Placenta praevia with bleeding	0.18%	0.13%	0.14%	0.06%	—	0.13%
Ovarian hyper stimulation	0.11%	0.10%	0.11%	0.14%	0.10%	0.10%
Bleeding before delivery, unspecified	0.07%	0.11%	0.09%	0.12%	0.19%	0.10%
Bleeding in early pregnancy, unspecified	0.11%	0.07%	0.08%	0.08%	0.14%	0.08%
Severe preeclampsia	0.06%	0.05%	0.08%	0.12%	0.38%^*∗∗*¶¶^	0.07%
Gastroenteritis and colitis	0.14%	0.04%	0.08%	0.02%	0.10%	0.06%
Vomiting in late pregnancy	0.08%	0.03%	0.07%	0.16%	0.00%^*∗∗*^	0.06%
Gestational diabetes	0.04%	0.02%	0.05%	0.20%^*∗∗*¶^	0.34%^*∗∗*¶¶^	0.05%
Proteinuria during pregnancy	—	0.03%	0.02%	0.12%^*∗*^	0.05%	0.03%
Essential hypertension, which complicates the pregnancy	—	0.01%	0.02%	0.06%	0.38%^*∗∗*¶¶^	0.03%
Nonspecified hypertension, which complicates the pregnancy	—	0.01%	—	0.02%	0.19%^*∗∗*^	0.01%

*∗* denotes significant difference between BMI between 20 and <25, ¶ between 25 and <30, # between 30 and <35. Single legends denote significance at level <0.05 and double legends denote significance at the <0.001 level.
